# Harnessing out-of-plane deformation to design 3D architected lattice metamaterials with tunable Poisson’s ratio

**DOI:** 10.1038/s41598-017-09218-w

**Published:** 2017-08-21

**Authors:** Tiantian Li, Xiaoyi Hu, Yanyu Chen, Lifeng Wang

**Affiliations:** Department of Mechanical Engineering, State University of New York at Stony Brook, Stony Brook, New York, 11794 USA

## Abstract

Auxetic materials exhibiting a negative Poisson’s ratio are of great research interest due to their unusual mechanical responses and a wide range of potential deployment. Efforts have been devoted to exploring novel 2D and 3D auxetic structures through rational design, optimization, and taking inspiration from nature. Here we report a 3D architected lattice system showing a negative Poisson’s ratio over a wide range of applied uniaxial stretch. 3D printing, experimental tests, numerical simulation, and analytical modeling are implemented to quantify the evolution of the Poisson’s ratio and reveal the underlying mechanisms responsible for this unusual behavior. We further show that the auxetic behavior can be controlled by tailoring the geometric features of the ligaments. The findings reported here provide a new routine to design architected metamaterial systems exhibiting unusual properties and having a wide range of potential applications.

## Introduction

Cellular structures are widely spread in natural systems, such as wood, human bone, and beaks of birds^1, 2^. They hold great promising applications, including aerospace, LED technologies, and automotive, due to their great specific mechanical properties. Recent studies show that by tailoring the architecture of the cellular structures, improved mechanical properties such as light weight^3, 4^, high energy absorption^5–10^, vibration control^11, 12^ and enhanced thermal performance^13–15^ can be simultaneously achieved. Along with these unusual properties and functionalities, recent advances in additive manufacturing techniques, for example 3D printing, have enabled the fabrication of cellular structures with well-defined topologies, thereby leveraging the possibilities to explore unprecedented properties in architected cellular structures. Here we use architected materials to emphasize that the unusual properties pertaining to the cellular structures strongly depend on the rationally designed architecture, rather than their compositions.

Recently, extensive research efforts have been devoted to investigating the unusual physical properties in architected cellular structures. Among these properties, architected cellular structures with a negative Poisson’s ratio (NPR) are of particular interest. These structures exhibit a counterintuitive mechanical response, as they will shrink (expand) laterally under uniaxial compression (stretch). Indeed, auxetic behavior has been reported in many 2D and 3D structures of natural systems, including cubic metals^16^, zeolites^17, 18^, natural layered ceramics^19^, silicon dioxides^20^, single-layer graphene^21, 22^, and 2D protein crystals^23^. Lakes has first designed and fabricated the first 3D polymeric foam with isotropic auxetic behavior^24^. Subsequently, a number of geometries have been proposed to achieve negative Poisson’s ratio. Among various architectures, it is worth noting that 2D related structures are the majority, including honeycomb with inverted cells^25, 26^, planar chiral lattices^27–29^, rigid rotating hexamers or squares^30–33^, origami-kirigami based metamaterials^34–39^ and hierarchical metamaterials with fractal cuts^40, 41^. Most of the auxetic effects in these 2D structures are due to the in-plane deformation, for example, the symmetric units with re-entrant angles^24, 42–47^ and asymmetric units^28, 32, 33, 48–52^ both rotate in the plane when deformed. Interestingly, soft kagome lattice based metamaterials with prescribed asymmetric units can achieve tunable negative Poisson’s ratio under a wide range of compressive strains by prescribing the pore units with designated pre-twisting angles^39^. And the transition between positive and negative depends on the symmetry of the ligaments and their arrangement, and the pre-twisting angles. Hierarchical kirigami-based metamaterials also indicate similar tunable Poisson’s ratios^38^.

Most of the theoretical and experimental investigations related to auxetic cellular materials are focused on the microstructures with straight ligament topologies. Recently, it has been theoretically shown that the auxetic behavior can be attained in hierarchically architected lattice consisting of horseshoe microstructures^53^. A nonstraight rib configuration for open-cell polyurethane foams has also recently been considered as a likely explanation for the existence of an unusual blocked-shape memory effect in auxetic open-cell polyurethane foams^54^. Moreover, our previous study reports a class of architected lattice metamaterials with sinusoidally shaped ligaments in the plane, which are highly stretchable with tunable negative Poisson’s ratios and vibration-mitigation capability^55^. Comparing with the comprehensive study of various 2D auxetic structures, fewer designs of synthetic 3D auxetic materials have been proposed. Among them, auxetic systems consisting of networks of buckliball^56^, chiral-like structures^57^, orthotropic laminated open-cell frameworks^58^ have been fabricated via 3D printing and very recently, a metallic 3D auxetic cellular structure consisting of cubic chiral unit cells has been fabricated via selective electron beam melting^59^. In all of these systems, however, the auxetic behavior is exhibited only in the limit of small strains, and the design of 3D auxetic systems capable of obtaining these unusual properties at large strains still remains a challenge^60, 61^.

Recent studies indicate that it is possible to harness out-of-plane deformation to achieve auxetic behavior. For instance, origami-based metamaterials^34, 35^, the dimpled plastic sheets^62^, and smooth curve sheet^63^ all exhibit negative Poisson’s ratio in a plane through out-of-plane deformation. Since these structures are all non-porous with heavy weight, the solid structures will suppress the large deformation amplitude. Here we report an architected lattice material system that exhibits tunable negative Poisson’s ratio over a wide range of applied uniaxial stretch, which is intrinsically governed by the out-of-plane deformation in the curved ligaments. We will demonstrate our design concept through integrative numerical simulation, analytical modeling, 3D printing, and experimental tests.

## 3D Planar Auxetic Metamaterials

**Figure 1:**
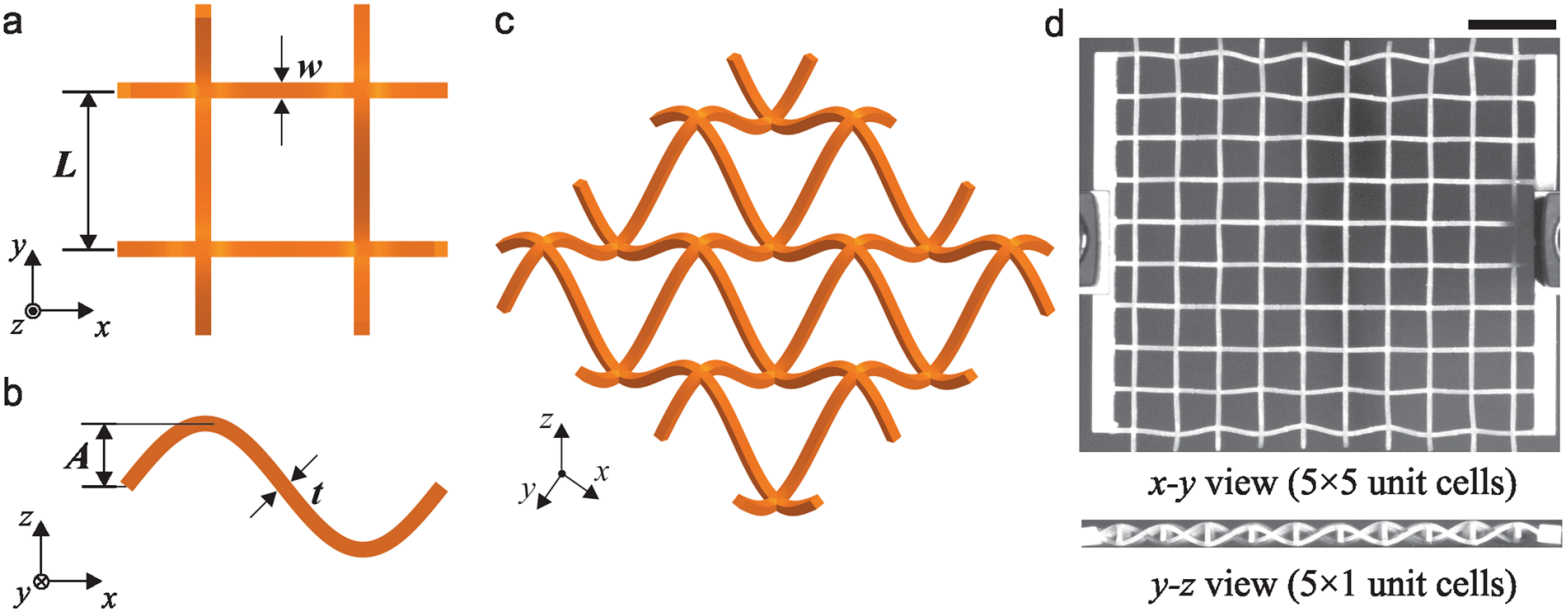
Overview of the proposed 3D planar auxetic metamaterials; (**a**) top and (**b**) side view of a unit cell, (**c**) schematic of the proposed construction, (**d**) photograph of a structure fabricated by 3D printing, comprising 5 × 5 × 1 unit cells in *x*, *y*, *z* coordinates. Scale bar: 2 cm.

The model structure presented here is a lattice metamaterial and consists of curved beam components made of the same material. Figure 1 shows an overview of the proposed structure, which is fabricated by an additive manufacturing technique (3D printing). A Cartesian coordinate system is used for the in-plane *x*-*y* and out-of-plane *x*-*z* coordinates. Figure 1(a) shows the top view of a unit cell of the structure which is a 2D regular square lattice consisting of the beams with the width, *w* and the length, *L*. We create the lattice system by replacing the regular straight beams with curved beams in the out-of-plane direction. The shape of the curved beams can be mathematically described as $z=A\,\sin (\pi x/L)$, where *A* is the wave amplitude and *t* is the thickness of the beam. Figure 1(c) illustrates the 3D lattice microstructure in 2 × 2 unit cells. The geometry of the structure is characterized by three dimensionless parameters: the normalized wave amplitude ratio, *A*/*L*, the normalized width of the beam, *w*/*L* and the normalized thickness of the beam, *t*/*L*. The proposed lattice metamaterials are fabricated using a multimaterial 3D printer (Objet Connex260, Stratasys). To ensure the stretchability of the cellular configuration, a rubber- like material, FLX9795-DM, is used as the constitutive (core) material for the sinusoidally shaped beams. Figure 1(d) shows a photograph of a real test specimen with 5 × 5 unit cells with *A*/*L* = 0.2, $w/L=t/L=0.1$. The dimensions of the structural units without side bars are 100 × 100 × 40 mm. Both ends of the specimens, in the *x*-axial direction, are added a rectangular rigid beam section with 5 mm width to provide a more uniform tensile displacement distribution.

## Results

To evaluate the auxetic behavior of the proposed architected lattice system, we perform uniaxial quasi-static tension tests on the 3D printed samples. In all the experiments, the displacement speeds are 0.1 mm/s (strain rate 0.001 s^−1^). In addition, the commercial finite element (FE) package ABAQUS/Standard (Simulia, Providence, RI) is used to investigate the response of the system under uniaxial tension. More details on the experimental and numerical techniques can be found in the Methods Section and Supplementary Information.

**Figure 2:**
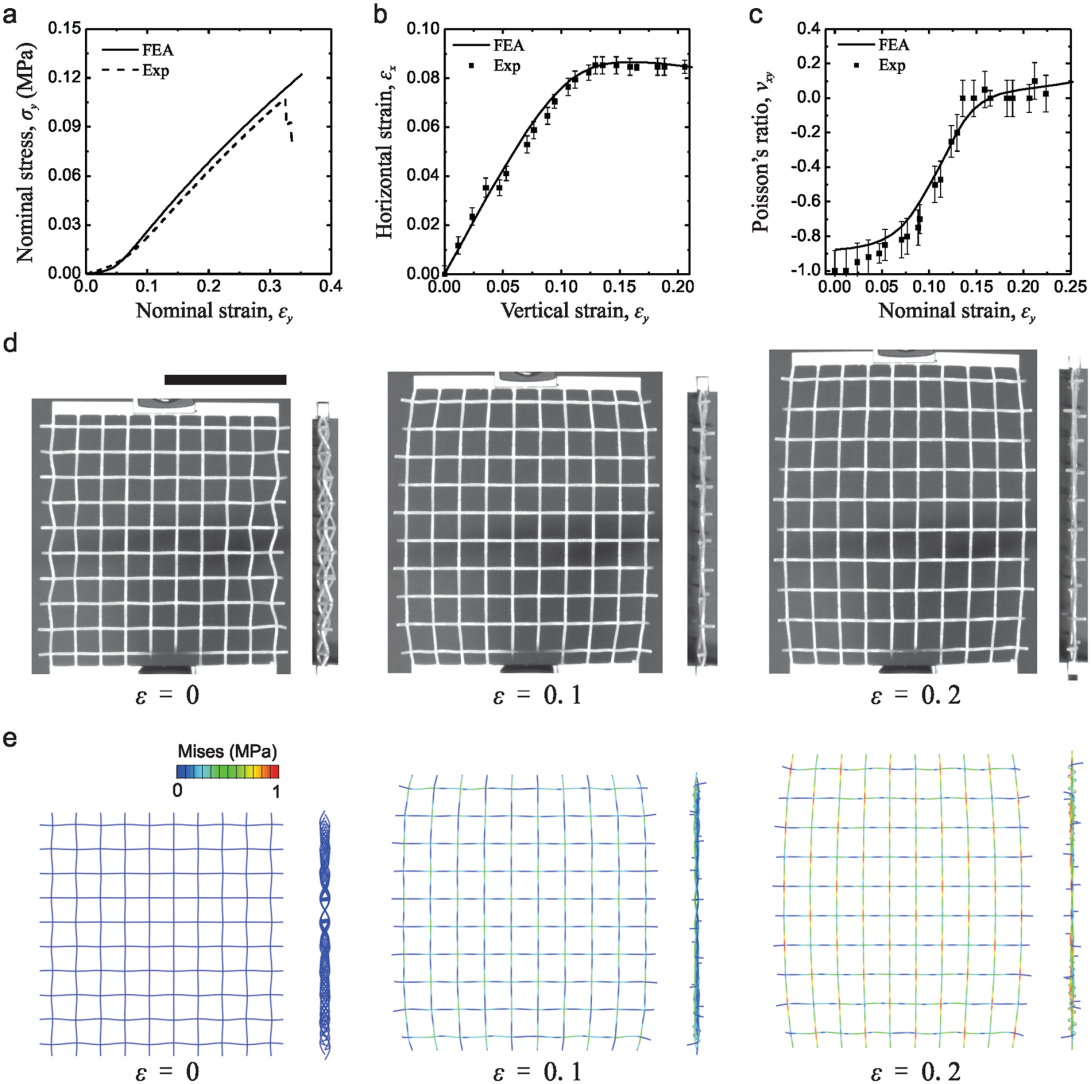
Experimental and FEA results of 3D planar auxetic metamaterials under uniaxial tensile tests: (**a**) nominal stress vs. nominal strain curve; (**b**) measured horizontal strain vs. vertical strain; (**c**) calculated incremental Poisson’s ratio curves as a function of nominal strain; (**d**) experimental and (**e**) simulation images at different levels of macroscopic strains: 0, 0.1, and 0.2. Scale bar: 5 cm.

The uniaxial tension testing results of the experiment and numerical analysis are summarized in Fig. 2. Figure 2(a) shows the stress-strain curve under tensile deformation up to the failure point. A good agreement between the experimental and numerical results is noted. When the applied strain is higher than 0.32, some beams start to break, leading to the drops in the stress-strain curves. These structures exhibit J-shaped stress-strain curves, which are very similar to the mechanical response of the lattice materials previously reported^55^. In Fig. 2(b) we present the relations between the horizontal strain and the vertical strain of the lattice material during uniaxial tension tests. The experimental data points are shown in comparison with the numerically determined solid lines and it can be seen that there is close agreement between the sets of results. The horizontal strain first increases during the initial linear elastic response of the periodic structures before it reaches around 0.1, afterward, it becomes independent of the vertical strain. Clearly, this structure has an auxetic behavior. Since the response of the structure is non-linear as shown in the stress-strain plot in Fig. 2(a), the incremental Poisson’s ratio *v* is calculated using the relations of horizontal and vertical strain. The experimental and numerical estimates of *v* are plotted as a function of nominal strain in Fig. 2(c). At small strain ( < 0.02), the numerically determined estimate of the Poisson’s ratio is approximately constant at ${\nu }_{num}=-0.875\pm 0.052$. The experimental result is slightly smaller than the numerical result with a value of ${\nu }_{\exp }=-0.950\pm 0.110$. This minor discrepancy between the simulated and experimental results is generated due to the experimental testing and digital image analysis. Unavoidable misalignments in the test setup tend to influence the measurement of the Poisson’s ratios since the lattice materials are relatively soft. When using digital image correlation, the calculation of strains using mark points is influenced by the accuracy of the location of mark points, leading to errors in the resulting Poisson’s ratio. With the increase of the stretching, the Poisson’s ratio gradually turns from negative to marginally positive. Figure 2(d) shows the specimen under deformation during the uniaxial tension test. It is clear that the lattice material expands transversally, indicating the presence of an auxetic behavior. The corresponding deformation images of numerical results are shown in Fig. 2(e). Good agreement between numerical simulations and experimental deformations in the lattice shapes can be observed. Moreover, we find that the applied uniaxial stretch causes out-of-plane shrinking (see side views in Fig. 2(d and e)). This is due to the mechanism of deformation of this lattice structure which will be discussed later. Furthermore, the 3D isometric view of the simulated deformed structure at different levels of macroscopic strains are shown in Fig. S4 (Supplementary Information) indicating how the 3D curved beams deform.

**Figure 3:**
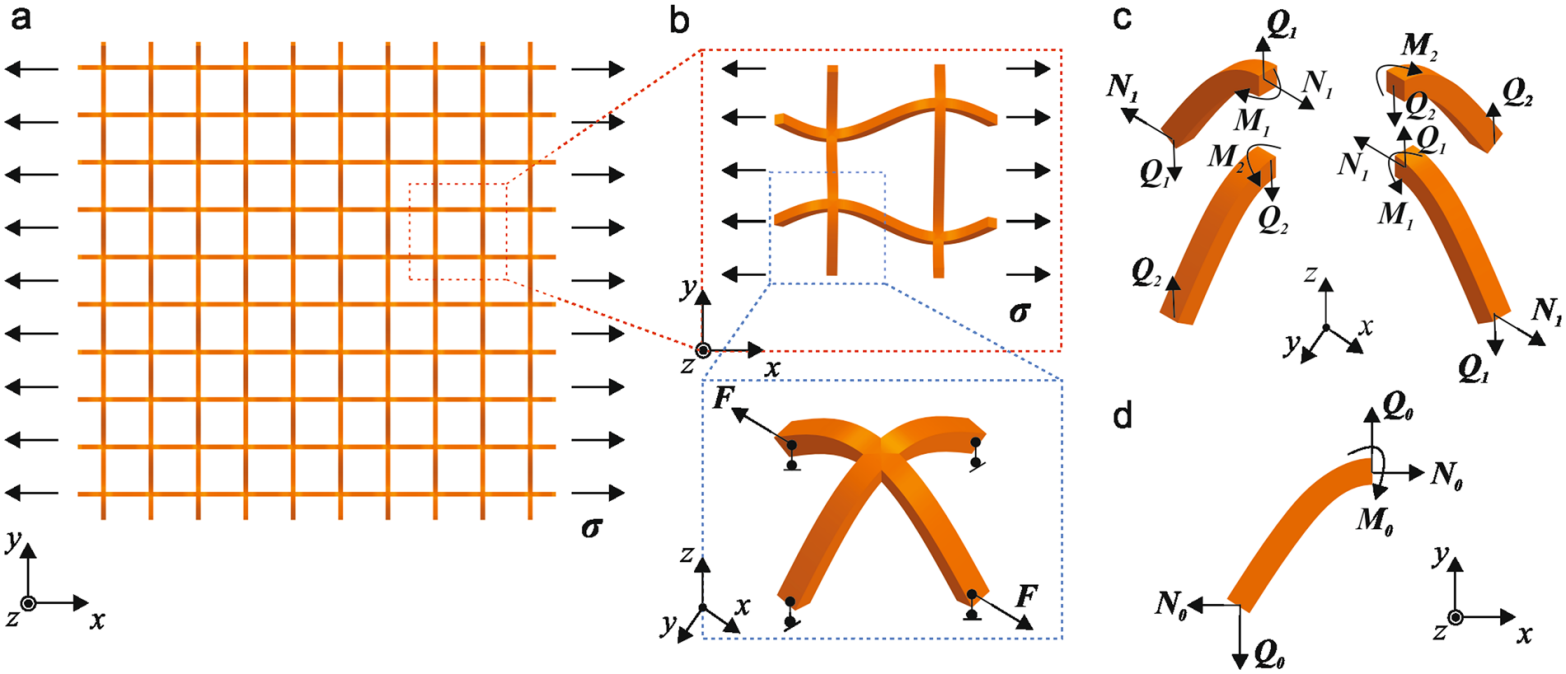
Schematics of the theoretical model of the 3D planar auxetic metamaterial subject to a uniform tensile stress along horizontal stretching: (**a**) the lattice materials with 5 × 5 unit cells under horizontal stretching is taken as an example to illustrate the model; (**b**) a representative unit cell is under horizontal stretching and due to the anti-symmetric one corner structure is analyzed; (**c**) free body diagrams of the corner structure; (**d**) a curved beam subject to axial forces, shear forces and a moment at the right end.

To quantitatively understand the mechanisms responsible for the auxetic behavior in the reported lattice system, we formulate an analytic model for the lattice system under uniaxial tension. The lattice materials with 5 × 5 unit cells under horizontal stretching are taken as an example to illustrate the model, as shown in Fig. 3(a). Due to the lattice periodicity, only a representative unit cell (in the red frame of Fig. 3(a)) is analyzed, as shown in Fig. 3(b). The unit cell consists of four corner structures, while each connected structure undergoes anti-symmetric deformations with respect to the central point of the unit cell. Only one corner structure is selected for the force analysis as shown in Fig. 3(c). The inner axial forces, shear force, and the moment at the ends are denoted by *N*_*i*_, *Q*_*i*_ and *M*_*i*_, respectively. The static equilibrium of the unit cell gives the relations among the inner forces and the external loading (normal stress *σ* along horizontal direction):

1 $${N}_{2}=0\,{\rm{and}}\,{N}_{1}=\sigma Lt.$$

The deformation compatibility requires that the joints of deformed beams should satisfy the following geometric relations:

2 $${h}_{1}={h}_{2}\,{\rm{and}}\,{Q}_{1}={Q}_{2}=Q.$$

where *h* is the height of the curved beam in an out-of-plane direction.

Since the geometry is symmetric with regard to the central point, we consider a curved beam ($z=A\,\sin (\pi x/L),0\le x\le 0.5L$) subject to inner forces *N*_0_, *Q*_0_ and a moment, *M*_0_ at one end on the *x*-*z* plane, as shown in Fig. 3(d). Based on the classic Elastic theory^53, 64–66^, if the effect of membrane deformation is neglected, we can introduce a model for small strain but finite rotation, which accounts for both bending and membrane deformation, to analyze the deformation and maximum strain in the microstructure. (See Supplementary Information for more details) By solving this model numerically, we can obtain the coordinates of the right end for the curved beam (*x*_*end*_, *z*_*end*_) as:

3 $${x}_{end}={f}_{1}({N}_{0},Q{}_{0})\,{\rm{and}}\,{z}_{end}={f}_{2}({N}_{0},Q{}_{0}),$$

where $h={z}_{end}$. Using Eqs (2) and (3), Eq. (1) the becomes

4 $${f}_{2}(\sigma Lt,Q)={f}_{2}(0,Q).$$

Therefore, for a given normal stress *σ*, the shear force *Q* can be solved directly. The deformation horizontal and vertical strain of this structure are:

5 $${\varepsilon }_{h}={f}_{1}(0,Q)/L\,{\rm{and}}\,{\varepsilon }_{v}={f}_{1}(\sigma Lt,Q)/L.$$

Full details of the theoretical model and mathematical operation can be found in the Methods and Supplementary Information.

**Figure 4:**
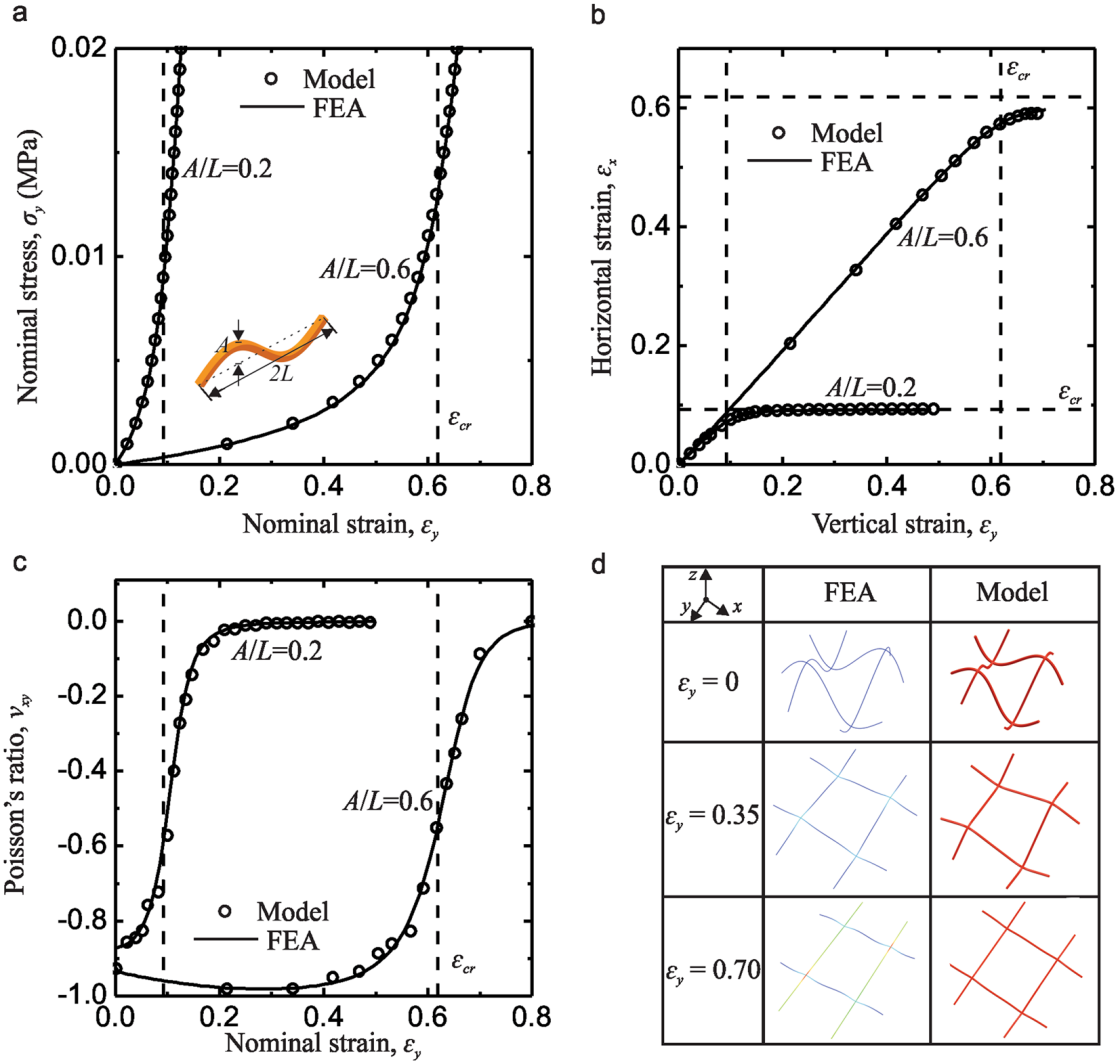
Theoretical and FEA results of 3D planar auxetic metamaterials under uniaxial tensile tests: (**a**) nominal stress vs. nominal strain curve; (**b**) measured horizontal strain vs. vertical strain; (**c**) calculated incremental Poisson’s ratio curves as a function of nominal strain; (**d**) Theoretical and (**e**) simulation images for the specimen with *A*/*L* = 0.6 at different levels of macroscopic strain: 0, 0.35, and 0.7. Here the dash lines present the critical strain which is the strain to fully extend the curved beam with sine wave.

In order to compare theoretical and simulated results, the mechanical response of the lattice materials under uniaxial stretching along vertical directions is presented in Fig. 4. All theoretical results agree reasonably well with the FEA calculations. Here we use two specimens consist of a representative unit cell with $w/L=t/L=0.1$ and *A*/*L* = 0.2 or 0.6, respectively. It is clearly that the specimen with *A*/*L* = 0.2 have a higher stress-strain curve within the small strains range compared with the specimen with *A*/*L* = 0.6, as shown in Fig. 4(a). The nominal stress-strain curve increases slowly at a low strain in a bending-dominated deformation mode, and increases rapidly after a critical strain, *ε*_*cr*_ due to a transition into stretching-dominated deformation mode. This critical strain is well presented by

6 $${\varepsilon }_{cr}=({\int }_{0}^{L}\sqrt{1+{(\frac{A\pi }{L}\cos (\frac{\pi x}{L}))}^{2}}dx)/L-1,$$

denoting the strain to fully extend the curved beam with sin wave, as marked by the dashed line in Fig. 4(a–c). Figure 4(b) shows that the relation of horizontal strain and vertical strain increases linearly at a low strain, but after a vertical critical strain, it approaches to a plateau which is the horizontal critical strain. It can be noticed that the vertical critical strain and horizontal critical strain are equal, which can be estimated as Eqn. 6. The calculated incremental Poisson’s ratios show negative values as −0.856 and −0.925 for the specimen with *A*/*L* = 0.2 and 0.6 respectively in Fig. 4(c). With the deformation mechanisms switching from bending to stretching at the critical strain, the Poisson’s ratio increase dramatically from negative to almost zero. The deformed configurations of the representative unit cell based on the theoretical prediction show very good accordance with the FEA results under different strain levels as shown in Fig. 4(d). It can be noted that the negative Poisson effect originates mainly from the dilatation of the lattice-shaped unit cell. Here, the beams deform from curved to straight in both directions which indicate a full expansion along the horizontal direction during vertical stretching. Therefore, our analytical model explains the reason for the auxetic behavior of this lattice structure and demonstrates a clear transition of deformation mode from bending-dominated to stretching-dominated behaviors in the uniaxial stretching.

**Figure 5:**
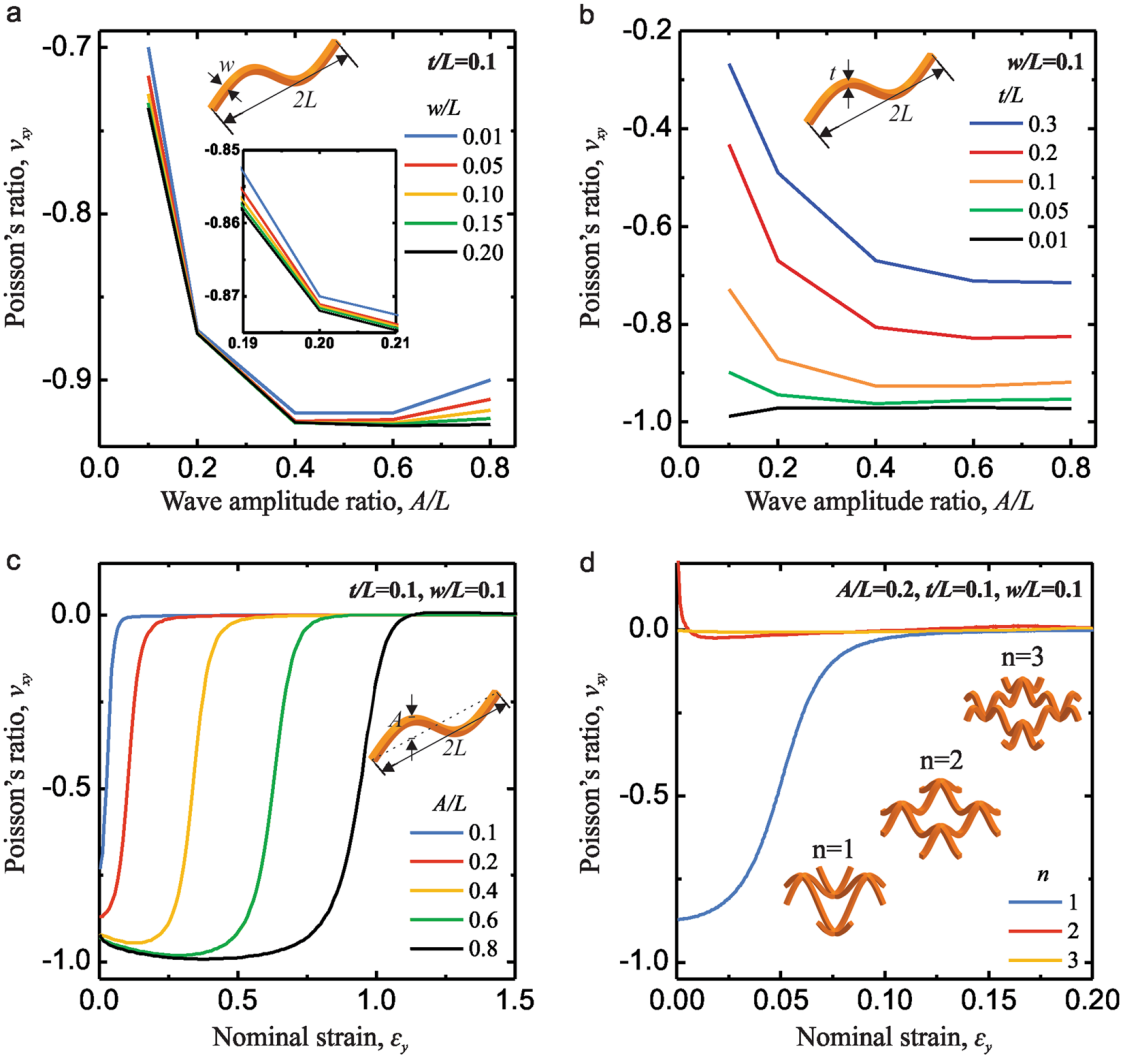
Effect of *A/L*, *w/L*, *t/L*, and *n* on the macroscopic Poisson’s ratio of the 3D planar metamaterials, *ν*_*yx*_. Evolution of *ν*_*yx*_ as a function of *A/L* is shown in (**a**) for five different values of *w/L* (assuming *t/L* = 0.1), in (**b**) for five different values of *t/L* (assuming *w/L* = 0.1). Evolution of *ν*_*yx*_ as a function of nominal strain is shown in (**c**) for five different values of *A/L* (assuming *t/L* = 0.1 and *w/L* = 0.1), and in (**d**) for three different values of *n* (assuming *A/L* = 0.2, *t/L* = 0.1 and *w/L* = 0.1).

Having demonstrated that the lattice metamaterials exhibit auxetic behavior under uniaxial tension at specific strain ranges, we now systematically investigate the effects of geometry parameters on the evolution of Poisson’s ratios, *v*. Because the Poisson’s ratio is highly strain-dependent, we select the values at very small strains (≤0.02) for each specimen. In Fig. 5(a), we report the evolution of *v* as a function of *A*/*L* for different values of *w*/*L*(0.01–0.20), while *t*/*L* is kept constant of 0.1. First, the results indicate that, as *A*/*L* increases, *v* initially drops, reaches a minimum value and then increases. Moreover, we find that the macroscopic Poisson’s ratio slightly decreases as the normalized width of the beam increases. In Fig. 5(b), we present the evolution of *v* as a function of *A*/*L* for different values of *t*/*L*(0.01–0.30), while *w*/*L* is kept constant of 0.1. Similarly, as *A*/*L* increases, *v* initially drop except the specimen with *t*/*L* = 0.01, reaches a minimum value and then keep almost constant. Furthermore, the macroscopic Poisson’s ratio increases as the normalized thickness of the beam increases. These mechanical responses are intrinsically controlled by the bending stiffness of the beams, ${E}_{S}I=1/12w{t}^{3}$, where *E*_*S*_ is the Young’s modulus of the material and *I* is the moment of inertia. In Fig. 5(c), we report the evolution of *v* as a function of nominal strain for different values of *A*/*L* (0.1–0.8), while *t*/*L* and *w*/*L* are both kept constant of 0.1. Interestingly, the transition strain for the Poisson’s ratio from negative to zero is proportional to the wave amplitude ratio. This is because large macroscopic stretching is needed to make the vertical beams with larger wave amplitude ratio straight. Moreover, the wave amplitude direction in our lattice materials is out-of-plane, which indicates that we can design lattice structures to achieve tunable Poisson’s ratio at extreme large stretching strains. Furthermore, we find that the responses of the system are affected by the wavelength number, *n*. In the previous lattice materials, the curved beam can be described as: $z=A\,\sin (n\pi x/L)$ with *n* = 1. It is noted that the auxetic behavior cannot be observed for *n* = 2 and 3, as seen in Fig. 5(d). Finally, these results suggest a reliable and versatile route to tailor the geometric features of lattice material with curved beams to achieve tunable mechanical properties especially negative Poisson’s ratio for specific mechanical applications.

## Discussion and Conclusions

Compared with conventional 2D lattice auxetics, the 3D architected lattice system reported here exhibits an auxetic behavior over a large range of applied strain. Theoretically, there is no limitation for the applied stretch strain since there is enough space to incorporate the curved ligaments in the out-of-plane direction. Moreover, the proposed architected lattice system is highly structural efficient in terms of the lightweight design, as compared with dimpled plastic sheets^62^ and origami based auxetics^34, 35^.

**Figure 6:**
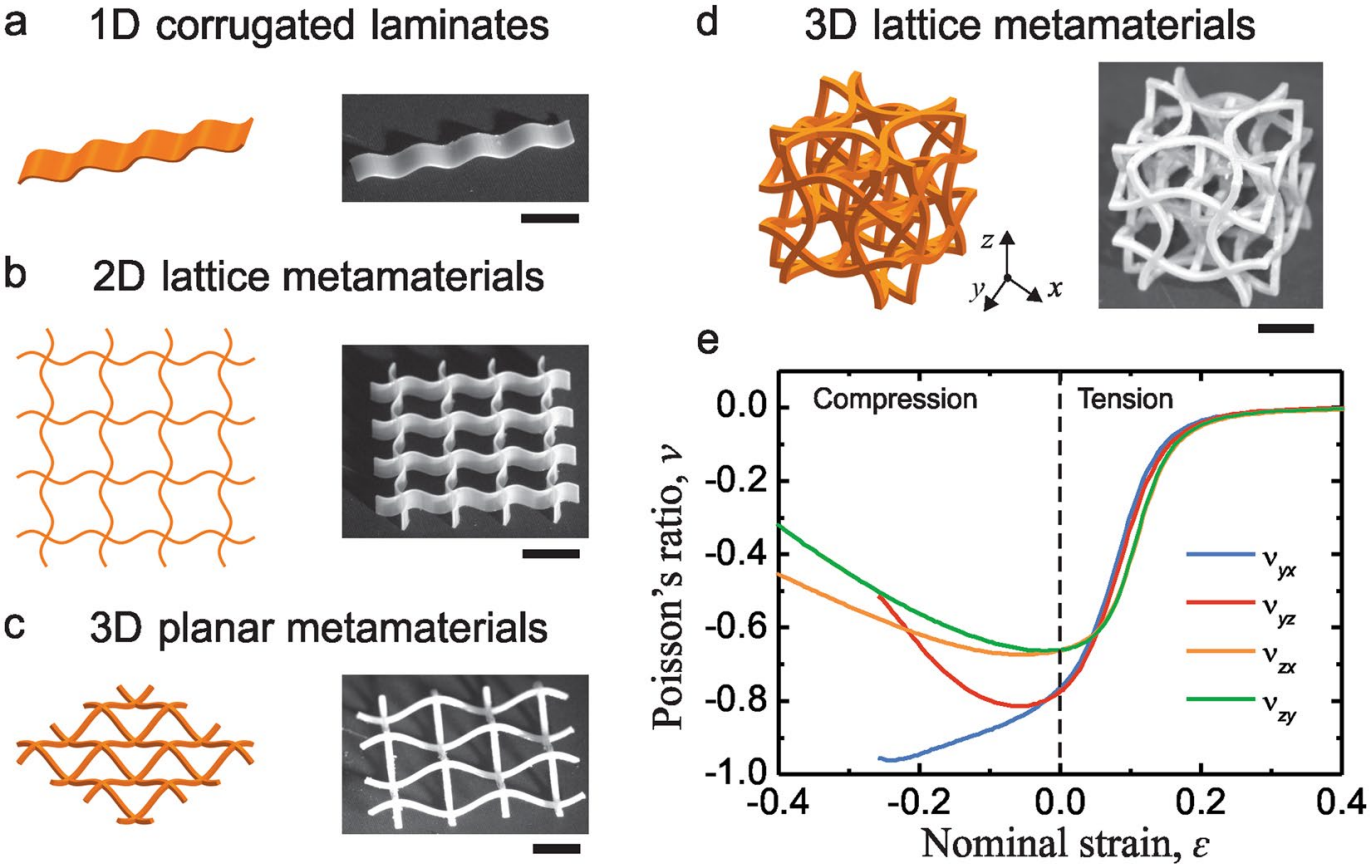
The system of lattice metamaterials with curved beams. (**a**) 1D corrugated beam; (**b**) 2D lattice metamaterial; (**c**) 3D planar metamaterial; (**d**) 3D lattice metamaterial. (**e**) The Poisson’s ratio as a function of nominal strain for the 3D lattice metamaterials under uniaxial compression and tension by FEM analysis. Because of the symmetry of the structures in *x* and *y* directions, we only show the results of loading in *y* and *z* directions. Scale bar: 1 cm.

Indeed, the design concept of replacing the straight beam with a curved beam can be extended to construct 1D, 2D, or 3D metamaterials (Fig. 6). Figure 6(a) presents the 1D corrugated laminates exhibiting the geometrically non-linear stiffness response which is crucial for applications with large deformation^67^. Figure 6(b) shows the 2D lattice metamaterials. Our previous report^55^ indicates that this systematic lattice metamaterial exhibits extreme Poisson’s ratio variations between −0.7 and 0.5 under large tensile deformations and remarkable broadband vibration-mitigation capability. Figure 6(c) presents the 3D planar metamaterials which have been studied in this paper. Comparing with 2D lattice metamaterials, our 3D planar metamaterials could demonstrate larger tensile deformation because of the structure extension in the 3^rd^ dimension. Moreover, we can use the structures of 3D planar metamaterials as a template to design a 3D lattice metamaterials, as shown in Fig. 6(d). This 3D cubic lattice structure consists of 2D chiral-like structures in the lateral surface and 3D planar metamaterials structures in the middle. Therefore, it exhibits tunable negative Poisson’s ratio not only under uniaxial compression but also uniaxial tension in *x*, *y* and *z* directions, as shown in Fig. 6(e). Furthermore, this material design strategy can be extended to 3D periodic lattice metamaterials, thus opening up the possibility of designing and analyzing novel materials with auxetic behavior.

In summary, we have demonstrated a fundamental new approach to generate 3D planar metamaterials with negative Poisson’s ratio by replacing the straight beams with curved beams in the out-of-plane direction. Through a combination of numerical analysis and experiments, we have shown that the Poisson’s ratio of the material system can be tuned and altered by designing the geometry of the curved beam structures. In particular, we present a theoretical study of nonlinear mechanical behavior in a class of lattice metamaterial with curved beams (sinusoidal shapes). This model can precisely predict the nonlinear stress-strain behavior and the Poisson ratio, as well as the deformed configurations under uniaxial deformation. Importantly, this material design strategy can be applied to create 1D, 2D to 3D metamaterials, providing insights into the development of classes of architected metamaterials with potential applications including energy absorption, tunable acoustics, vibration control, responsive devices, soft robotics, and stretchable electronics.

## Methods

### Materials

A rubber-like flexible material (FLX9795-DM, with material properties presented in Supplementary Information is used to 3D print the experimental specimen. The material properties are measured through tensile testing on dog-bone specimens based on ASTM D412. The basic properties of this material are characterized by a Young’s modulus of *E* = 5.5 MPa, Poisson’s ratio *v* = 0.37, and density *ρ* = 1157 kg/m^3^ in the undeformed configuration and they are obtained by fitting the response under uniaxial tension of the bulk material (see Supplementary Information for more details).

### Fabrication using 3D printing

The specimena are fabricated using Objet Connex260 multi-material 3D printer (Stratasys). Lattice metamaterials have overall dimensions of 100 mm × 100 mm and is composed of 10 × 10 unit cells. Each beam ligament has a thickness of 1 mm. Two rigid beams are also added to the top and bottom of the lattice structures to improve the connection alignment (see Supplementary Information for more details). Within the limitation of 3D printing technology, the layer orientation is found to influence the mechanical properties of the material. Therefore, all the specimens are printed along the same orientation on the printer build platform. The as-fabricated specimens are kept at room temperature for 7 days to allow for the saturation of the curing.

### Mechanical testing

Uniaxial tension tests are performed using a MTS mechanical tester (C43) with a 1 kN load cell. All experiments are conducted in a quasi-static regime with a constant strain rate of 0.001 s^−1^. The load-displacement curves measured from the uniaxial tensile tests are then transferred into nominal stress-strain behaviors based on the measured dimensions of the specimens. In order to calculate the Poisson’s ratio at each level of applied tension, the photographs of deformed configurations of the specimen are recorded using a digital camera. (See Supplementary Information for more details).

### Numerical simulations

The numerical analyses are performed using the commercial FE package ABAQUS/Standard (Simulia, Providence, RI). The response of finite-size (10 × 10 unit cells) lattice materials is investigated under uniaxial tension throughout this work. All models are generated by beam elements (ABAQUS hybrid element type B22H) and the accuracy of the mesh is insured by a mesh refinement study. In addition, geometric nonlinearity is considered to represent the large deformation of the structure. The models are subjected to uniaxial static tension along vertical directions while the horizontal contractions are monitored.

## Electronic supplementary material

Supplementary Information
