# Supplementary material for: Harnessing out-of-plane deformation to design 3D architected lattice metamaterials with tunable Poisson’s ratio
Source: Sci Rep. 2017 Aug 21;7:8949. doi: 10.1038/s41598-017-09218-w (PMC5566377; doi:10.1038/s41598-017-09218-w)
Supplement: Supplementary file 1 — Supplementary Information [file 41598_2017_9218_MOESM1_ESM.pdf]

Supplementary Information for

Harnessing out-of-plane deformation to design 3D architected lattice metamaterials with tunable Poisson's ratio

Tiantian Li,[1] Xiaoyi Hu, [1] Yanyu Chen[1] and Lifeng Wang[1]*

[1]*Department of Mechanical Engineering, State University of New York at Stony Brook, Stony Brook, New York 11794, USA*

**Correspondence Author. Tel: +1 631 632 1182; E-mail: lifeng.wang@stonybrook.edu*

S1. Experimental details

S1.1. Mechanical properties of constitutive material

The material properties of the FLX9795-DM rubber-like material were obtained by measuring the mechanical response of the 3D printed dogbone specimens. The experimental setup is shown in Figure S1 (a). For the dogbone specimen, speckles were sprayed on the samples using a spray paint for digital image correlation (DIC) measurements. The deformation and local strain contours of the samples were tracked by using DIC (Vic-2D, Correlated Solution) to calibrate the nominal stress-strain curves and to obtain the Poisson's ratio of the constitutive material. Figure S1 (b) shows the measured stress-strain curves (true and engineering strain) under uniaxial tension. According to ASTM 412, the basic properties of FLX9795-DM are characterized by a Young's modulus of $E = 5.5$ MPa, Poisson's ratio $\nu = 0.37$, and density $\rho = 1157$ kg/m^3. Here the Young's modulus is obtained from the measured stress-strain curve of dogbone specimen. Poisson's ratio of dogbone specimen is calculated by following the method in S 1.2. The density is obtained by averaging the densities of five dogbone specimens.

S1.2. Calculation of Poisson's ratio

To quantify the deformation taking place in the lattice materials during the experiments an image processing software (ImageJ 1.49 q) was used to determine the intersection points in the specimen. The deformation near the four edges of the specimen was strongly affected by boundary conditions. Therefore, we focused on the central 30% of the specimens to avoid Saint Venant effects from the edges, as shown in Figure S2 (b). The intersection points at the corners of the chosen area were determined as $\left(X_{i,j}, Y_{i,j}\right)$ in the undeformed and $\left(x_{i,j}, y_{i,j}\right)$ in the deformed state, respectively. The row and the column indices vary between $1 \le i \le 4$ and $1 \le j \le 4$. For each unit cell, the horizontal and vertical distances were calculated from the coordinates $\left(x_{i,j}, y_{i,j}\right)$, i.e., $\Delta x_{i,j} = x_{i,j+1} - x_{i,j}$ and $\Delta y_{i,j} = y_{i+1,j} - y_{i,j}$. Prior to the application of the tensile loading, we assessed the deformations in the undeformed state, i.e., $\Delta X_{i,j} = X_{i,j+1} - X_{i,j}$ and $\Delta Y_{i,j} = Y_{i+1,j} - Y_{i,j}$. A schematic diagram of the central region of the lattice structure under consideration with the definitions of $\Delta x_{i,j}$, $\Delta y_{i,j}$, $\Delta X_{i,j}$ and $\Delta Y_{i,j}$ is shown in Figure S2 (b). The local homogenized values of the engineering strain for each unit cell were determined as:

$$\varepsilon_{xx,i,j} = \Delta x_{i,j} / \Delta X_{i,j} - 1 \text{ and } \varepsilon_{yy,i,j} = \Delta y_{i,j} / \Delta Y_{i,j} - 1. \quad \text{(S1)}$$

The local values of the engineering strain were then used to calculate local values of the Poisson's ratio as:

$$v_{i,j} = -\frac{\varepsilon_{xx,i,j}}{\varepsilon_{yy,i,j}} . \quad \text{(S2)}$$

In our case, the loading is nonlinear, the incremental Poisson's ratio, $v_{in,i,j}$, should be calculated as:

$$v_{in,i,j} = \frac{\Delta v_{i,j}}{\Delta \varepsilon_{yy,i,j}}. \tag{S3}$$

Finally, the ensemble average Poisson's ratio of the nine central unit cells under consideration was computed as $v_{yx} = \langle v_{in,i,j} \rangle$.

S2. Numerical simulations

The numerical simulations related to the mechanical response of the lattice metamaterials are conducted using commercial FE package ABAQUS/Standard (Simulia, Providence, RI). We have used models with 5 × 5 unit cells in all the simulations (Figure S3 (a) and (b)). All models are generated by beam elements (ABAQUS hybrid element type B22H) and meshed after a convergence test. In addition, geometric nonlinearity is considered to represent the large deformation of the structure.

Here, in our simulation, we use two types of boundary conditions. To simulate the experimental conditions in the numerical analysis, a uniaxial displacement loading is applied on the top surface, while the bottom is fixed along both the *x* and *y* directions (Figure S3 (a)). Under this boundary condition, the simulated results agree very well with the experimental results, as seen in Figure 2. To simulate the ideal conditions with avoiding Saint Venant effects from the edges, a uniaxial displacement loading is applied on the top surface, while the bottom surface is fixed along the *y* direction and the left surface is fixed along the *x* direction (Figure S3 (b)). Note that the periodic boundary conditions derived above are validated by comparison with analytical expressions and they agree very well, as shown in Figure 4. Therefore, for parametric analysis, the periodic boundary conditions are applied. Material model is critical to achieve better agreement between numerical simulation, experiment, and analytical model. Here we use a linear elastic model with a Young's modulus of $E = 5.5$ MPa, Poisson's ratio $v = 0.37$.

The postprocessing of the Poisson's ratio was focused on the unit cell in the central region to avoid finite size and boundary conditions effects (Figure S3 (c)). The Poisson's ratio can be calculated from the incremental ratio of the nominal strain in the horizontal edge and vertical edge of the rectangular unit cell. Specifically, we first calculated the average displacement component of the four edges, from which the strain along horizontal and vertical directions can be calculated as:

$$\varepsilon_x = \frac{\bar{u}^R - \bar{u}^L}{2l} \text{ and } \varepsilon_y = \frac{\bar{v}^T - \bar{v}^B}{2l}. \tag{S4}$$

In equation (S4) $\bar{u}$ and $\bar{v}$ indicate the average horizontal and vertical displacement components respectively; *R*, *L*, *T*, and *B* denote the right, left, top, and bottom edges of the unit cell, respectively (Figure S3 (c)). Finally, the incremental Poisson's ratio is calculated as:

$$\nu_{yx} = -\frac{\Delta\varepsilon_x}{\Delta\varepsilon_y}. \tag{S5}$$

S3. Analytical model

To describe the curved beam on a 2-D reference configuration, the undeformed length element dS after deformation becomes the deformed length element ds. The coordinate of the end point (X, Y) in the un-deformed state deforms to (x, y) shown as Figure S5 (b). At the un-deformed state, the tangent slope angle at (X, Y) is denoted by α. At the deformed state, the tangent slope at (x, y) is denoted by θ. The deformation at (X, Y) is denoted by (u, v) where u is the horizontal displacement, and v is the vertical displacement. Hence

$$x = X + u, \; y = Y + v. \tag{S6}$$

The rotation angle φ can be found by

$$\varphi = \theta - \alpha. \tag{S7}$$

Since the strain at the centroid axis is defined by $\varepsilon = (ds - dS)/dS$, or

$$ds = (1+\varepsilon)dS\,. \tag{S8}$$

As in the case of in-extensional curved beam, $\varepsilon = 0$. For any length element dS, there is a corresponding radius of curvature R, such that

$$dS = Rd\alpha\,. \tag{S9}$$

Here the radius of curvature R does not have to be a constant.

For the deformed length element ds, the corresponding radius of curvature is denoted by r, i.e.

$$ds = rd\theta\,. \tag{S10}$$

For a linear elastic material with the Young's modulus E_S, integration of the above equation then gives the axial force N, shear force Q and bending moment M (per unit thickness, Fig 2 (d)) in the beam as

$$N = E_s A\varepsilon \text{ and } M = E_s I(1+\varepsilon)\frac{d\varphi}{ds}\,. \tag{S11a, b}$$

The equilibrium equations are

$$\frac{dM}{ds} - Q = 0\,,\ \frac{dQ}{ds} - N\frac{d\theta}{ds} = 0\,,\text{ and } \frac{dN}{ds} + Q\frac{d\theta}{ds} = 0\,. \tag{S12a, b, c}$$

For the loading condition shown in Figure S3 (a-b), the axial and shear forces are

$$N = N_0\cos\theta + Q_0\sin\theta \text{ and } Q = N_0\sin\theta - Q_0\cos\theta\,, \tag{S13}$$

which satisfy Eqs. (S12b) and (S12c). Its substitution into Eq. (S12a), together with Eq. (S11), gives

$$\frac{d^2\varphi}{d^2S} = (1+\frac{N_0\cos\theta + Q_0\sin\theta}{E_s A})(\frac{N_0\sin\theta - Q_0\cos\theta}{E_s I})\,. \tag{S14}$$

In our case, the shape of the sinusoidal beams can be mathematically described as $Z = A\sin(\pi X / L) = A\sin(BX)$, where A is the wave amplitude and $B = \pi / L$. The length of sinusoidal beam is given by

$$S = \int_0^{L/2} \sqrt{1+(Z')^2}\,dX = \int_0^{L/2} \sqrt{1+(AB\cos(BX))^2}\,dX\ , \tag{S15}$$

which gives that

$$dS = \sqrt{1+(AB\cos(BX))^2}\,dX\ . \tag{S16}$$

And for the sinusoidal curve, the radius of curvature R is given by

$$\alpha = \arctan(Z') = \arctan(AB\cos(BX)) = f(X)\,. \tag{S17}$$

From Eq. (11), the derivation $d\varphi / dS$ could be denoted as

$$\frac{d\varphi}{dS} = \frac{d\varphi}{dX}\frac{dX}{dS} = \frac{1}{\sqrt{1+(AB\cos(BX))^2}}\frac{d\varphi}{dX} = n(X)\frac{d\varphi}{dX} \tag{S18}$$

Similarly, the derivation $d^2\varphi / dS^2$ could be denoted as

$$\frac{d^2\varphi}{dS^2} = n^2(X)\frac{d^2\varphi}{dX^2} + n'(X)n(X)\frac{d\varphi}{dX}\,, \tag{S19}$$

Substitute Eq. (S17) to Eq. (S7) and derivation gives the expression of $d\varphi/dX$ and $d^2\varphi/dX^2$ as

$$\frac{d\varphi}{dX} = \frac{d\theta}{dX} - \frac{d\alpha}{dX} = \frac{d\theta}{dX} - f'(X)\,, \tag{S20a}$$

$$\frac{d^2\varphi}{dX^2} = \frac{d^2\theta}{dX^2} - f''(X)\,. \tag{S20b}$$

Substitute Eq. (17a, b) into Eq. (16), it becomes

$$\frac{d^2\varphi}{dS^2} = n^2(X)\frac{d^2\theta}{dX^2} + n'(X)n(X)\frac{d\theta}{dX} + (-n'(X)n(X)f'(X) - n^2(X)f''(X))\,. \tag{S21}$$

Then substitute Eq. (18) to Eq. (11) and simply gives

$$h(X)\frac{d^2\theta}{dX^2}+g(X)\frac{d\theta}{dX}+m(X)=H(\theta),\tag{S22a}$$

where $h(X)$, $g(X)$, $m(X)$ and $H(\theta)$ are defined as

$$h(X)=(1+(AB\cos(BX))^2)^{-1},\tag{S22b}$$

$$g(X)=A^2B^3\sin(BX)\cos(BX)(h(X))^2,\tag{S22c}$$

$$m(X)=-AB^3\cos(BX)(-1-2A^2B^2+(AB)^2\cos(2BX))h^3(X)),\tag{S22d}$$

$$H(\theta)=(1+\frac{N_0\cos\theta+Q_0\sin\theta}{E_sA})(\frac{N_0\sin\theta-Q_0\cos\theta}{E_sI}).\tag{S22e}$$

For the deform mode as shown in Figure S3 (a), the vanishing bending moment at the left end can be written as $d\varphi/d\alpha=0$, which gives the first boundary condition as

$$\frac{d\theta}{dX}\Big|_{X=0}=0\tag{S23}$$

And at the right end, the tangent slope angle keeps unchanged (because of the symmetric geometry), which can be evaluated as

$$\theta\Big|_{X=L/2}=0\tag{S24}$$

And at the right end, the deformed coordinates are $x=x_{end}$ and $z=z_{end}$ (because of the anti-symmetric geometry), which can be evaluated by integrating $dx=\cos\theta ds$ and $dz=\sin\theta ds$ from the left end to the right end, i.e.,

$$x_{end}=\int_0^{l/2}\cos\theta(1+\frac{N_0\cos\theta+Q_0\sin\theta}{E_SA})h^{-1/2}(X)dX,\tag{S25a}$$

$$z_{end}=\int_0^{l/2}\sin\theta(1+\frac{N_0\cos\theta+Q_0\sin\theta}{E_SA})h^{-1/2}(X)dX.\tag{S25b}$$

By solving Eqs. (S23) and (S24), both the deformed angles and coordinates at the two ends, i.e., θ_{end}, x_{end} and z_{end}, can be. We numerically solve this problem in Matlab by using Runge-Kutta method and several search methods. In other words, the following constitutive relation can be obtained:

$$x_{end} = f_1(N_0, Q_0) \text{ and } z_{end} = f_2(N_0, Q_0). \tag{S26}$$

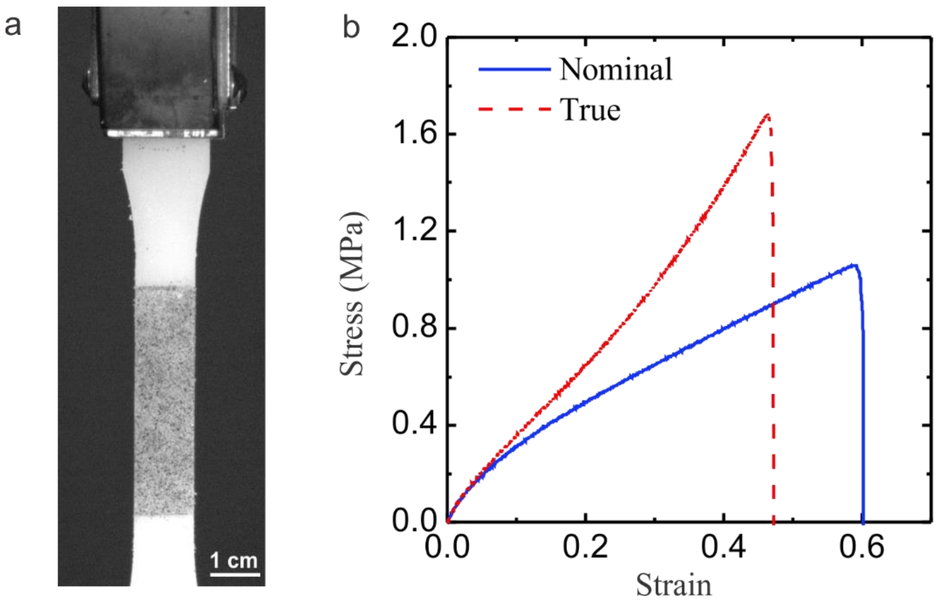

Figure S1. (a) 3D printed dogbone specimen under uniaxial tension. **(b)** Measured stress-strain relation of the dogbone specimen.

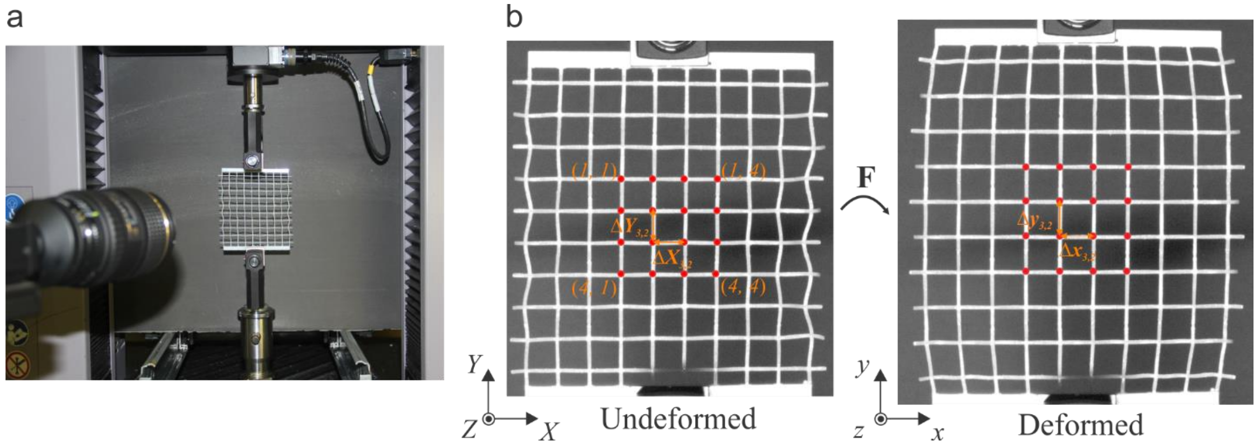

Figure S2. (a) Experimental setup of the lattice specimen under uniaxial tension. **(b)** Calculation of Poisson’s ratio.

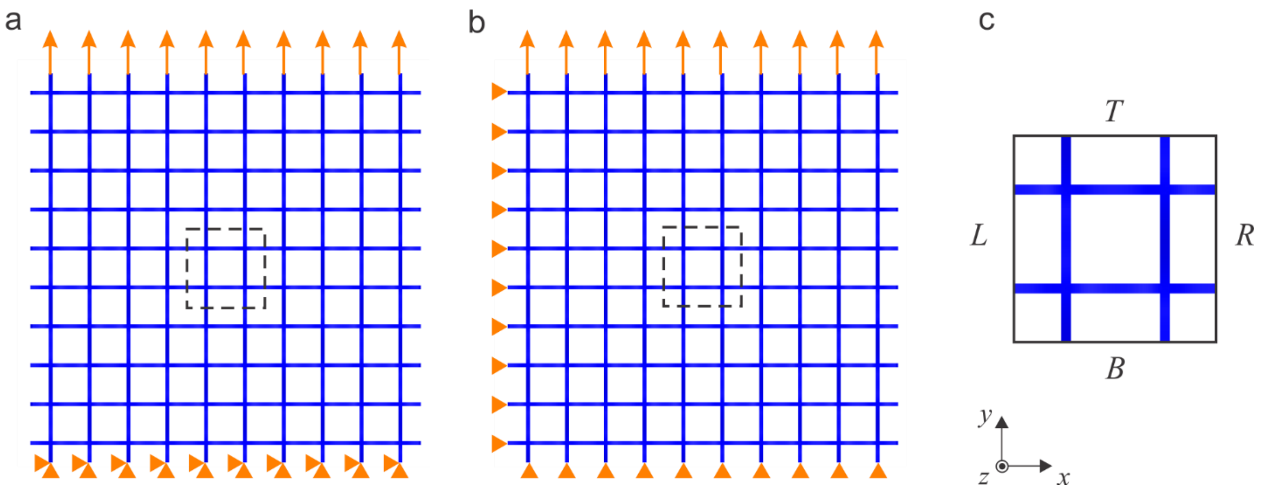

Figure S3. (a) FE model with boundary conditions for comparison with the experimental test. **(b)** FE model with periodic boundary conditions.

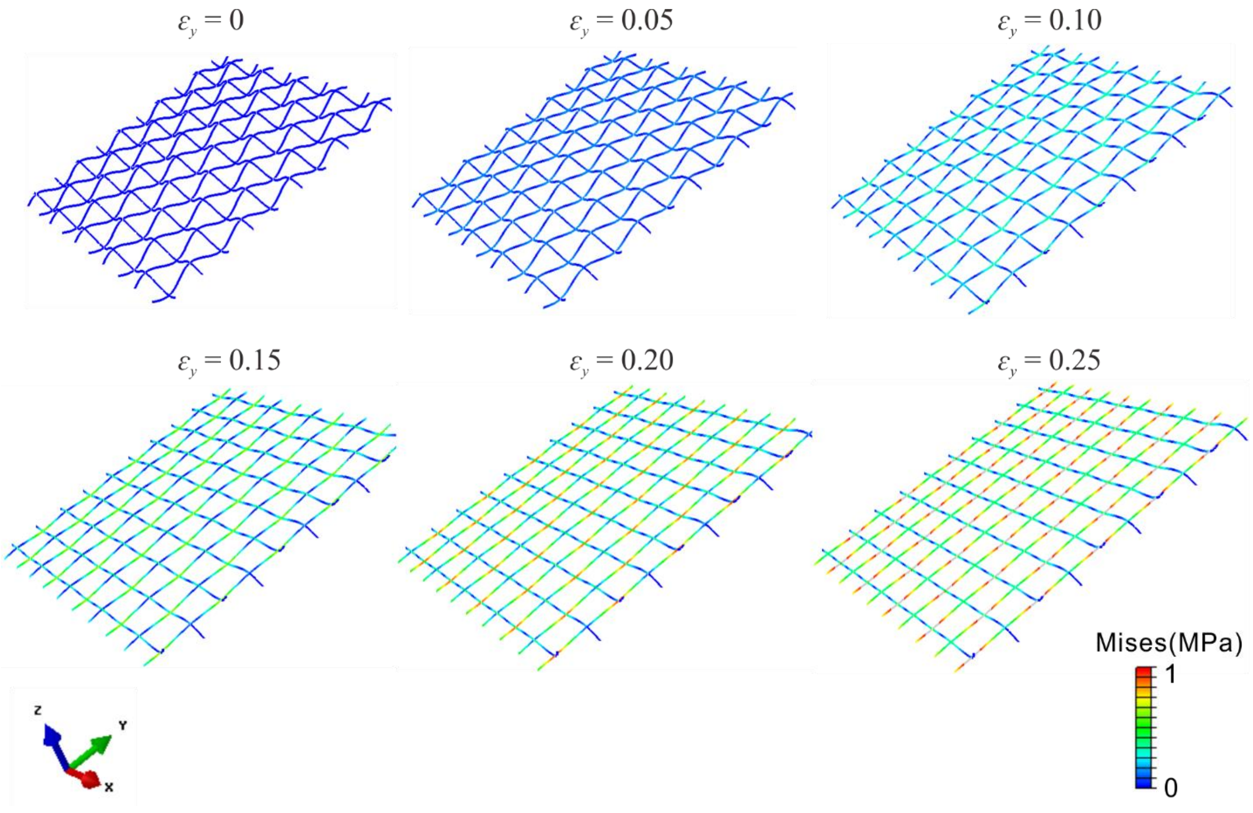

Figure S4. 3-D isometric view of the simulated deformed structure at different levels of macroscopic strains.

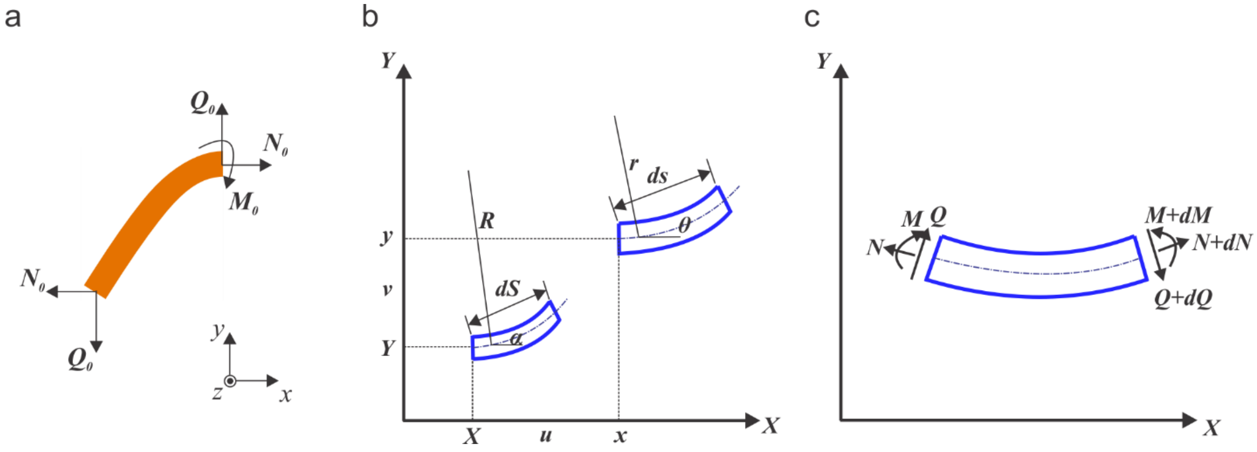

Figure S5. (a) A curved beam subject to axial forces, shear forces and a moment at the right end; **(b)** deformation of a unit length element; **(c)** sign conventions of forces and moment.
